# Analysis of *Cucurbita ficifolia* (Cucurbitaceae) chloroplast genome and its phylogenetic implications

**DOI:** 10.1080/23802359.2021.1959440

**Published:** 2021-09-24

**Authors:** Tao Zhang, Jun-Jun Xie, Jie Zhang, Zheng-An Yang, Xue Li, Shui-Lian He

**Affiliations:** College of Horticulture and Landscape, Yunnan Agricultural University, Kunming, China

**Keywords:** *Cucurbita ficifolia*, chloroplast genome, phylogenetic analysis

## Abstract

The figleaf gourd (*Cucurbita ficifolia* Bouché), is a member of the Cucurbitaceae. Figleaf gourd genotypes are exclusively used as a rootstock for cucumber owing to their high physiological compatibility with cucumber. In this study, the complete chloroplast (cp) genome of *C. ficifolia* was assembled. The cp genome of *C. ficifolia* was 157,631 bp in length, it consists of a pair of inverted repeats (IRa and IRb) regions (25,638 bp) separated by the large single-copy (LSC, 88,211 bp) and small single-copy (SSC, 18,144 bp) regions. The cp genome encodes 111 unique genes, including 80 protein-coding genes, 27 transfer RNA genes, and four ribosomal RNA genes. The overall GC content of *C. ficifolia* cp genome was 37.2%. The phylogenetic tree of Cucurbitaceae showed that *C. ficifolia* was clustered into genus *Cucurbita* and the bootstrap value is 100%.

The figleaf gourd (*Cucurbita ficifolia* Bouché), which is synonymous with black-seed gourd, is a member of the Cucurbitaceae. It most likely originated in Central Mexico, subsequently spreading to South America, with an ecological preference for highland areas (Arriaga et al. 2006; Kim et al. 2010). The tender immature fruits, mature seed and young leaves of figleaf gourd are edible. Figleaf gourd genotypes are exclusively used as a rootstock for cucumber in Far Eastern and Western countries owing to their high physiological compatibility with cucumber (Roxas 1994; Lee and Oda 2003). While the figleaf gourd is highly tolerant to low temperature and salinity, it is susceptible to biotic stresses, such as nematodes and a *Fusarium* spp. (Lee and Oda 2003). To date, no genome information are available despite the potential value of this plant as a genetic resource for squash breeding. In the present study, the whole chloroplast (cp) genome of *C. ficifolia* was sequenced, assembled and annotated. Understanding the chloroplast genome information of this species may provide valuable guidelines for squash breeding.

The fresh leaves of *C. ficifolia* were collected from wild field of Mile county, Yunnan province of China (24.13°N, E103.31°E). The voucher specimen (Cuf6) was deposited at Laboratory of College of Horticulture and Landscape, Yunnan Agriculcural University (Voucher specimen: YNAU003512). Total genomic DNA was isolated from fresh leaves using a DNeasy Plant Mini Kit (QIAGEN, Valencia, California, USA) according to the manufacturer’s instructions to construction chloroplast DNA libraries. The Illumina sequencing was conducted by Shanghai Genesky Biotechnologies Inc. (Shanghai, China). Resultant clean reads were assembled using GetOrganelle pipeline (Jin et al. 2018, https://github.com/Kinggerm/GetOrganelle). The genome was automatically annotated by using the CpGAVAS pipeline (Liu et al. 2012) and start/stop codons and intron/exon boundaries were adjusted in Geneious R11.0.2 (Kearse et al. 2012). All the contigs were checked against the reference genome of *C. maxima* (NC_036505) and *Cucurbita pepo* (NC_038229). The raw data of sequence and annotation results were submitted to NCBI, under the accession number MW801448 and SRA number SRR14055461.

The complete cp genome sequence was 157,631 bp in length. It was the typical quadripartite structure and contained two short inverted repeat (IRa and IRb) regions (25,638 bp) which were separated by a small single copy (SSC) region (18,144 bp) and a large single copy (LSC) region (88,211 bp). The cp genome encodes 111 unique genes, including 80 protein-coding genes (PCGs), 27 transfer RNA (tRNA) genes, and four ribosomal RNA (rRNA) genes. Eighteen gene species are partially or completely duplicated, including seven PCGs, (*ndhB*, *rpl2*, *rpl23*, *rps12*, *rps7*, *ycf15*, *ycf2*), seven tRNAs (*trnA-TGC*, *trnI-CAT*, *trnI-GAT*, *trnL-CAA, trnN-GTT*, *trnR-ACG, trnV-GAC*), all four rRNAs (4.5S, 5S, 16S, 23S rRNA). The GC content of the cp genome was 37.2%, while the corresponding values of LSC, SSC, and IR regions was 34.9%, 31.6%, and 43.0%, respectively.

The 48 chloroplast genome sequences of Cucurbitaceae were downloaded from GenBank and aligned with *C. ficifolia* using MAFFT (Katoh and Standley 2013) in Geneious R11.0.2 (Kearse et al. 2012). To resolve its phylogenetic placement within the family Cucurbitaceae, the maximum likelihood (ML) phylogeny tree was reconstructed using IQtree (Lam-Tung et al. 2015). *Begonia guangxiensis* (NC_046385), *Begonia pulchrifolia* (NC_045096), and *Begonia versicolor* (NC_047450) from Begoniaceae were selected as outgroups. The topology of the phylogenetic tree showed that the species of *C. ficifolia* was clustered into genus *Cucurbita* (Figure 1), the genus *Cucurbita* is a monophyletic group and the bootstrap value is 100%. The complete cp genome information reported in this study will be a valuable resource for future studies of the species’ genetic diversity, and breeding research of squash.

**Figure 1.**
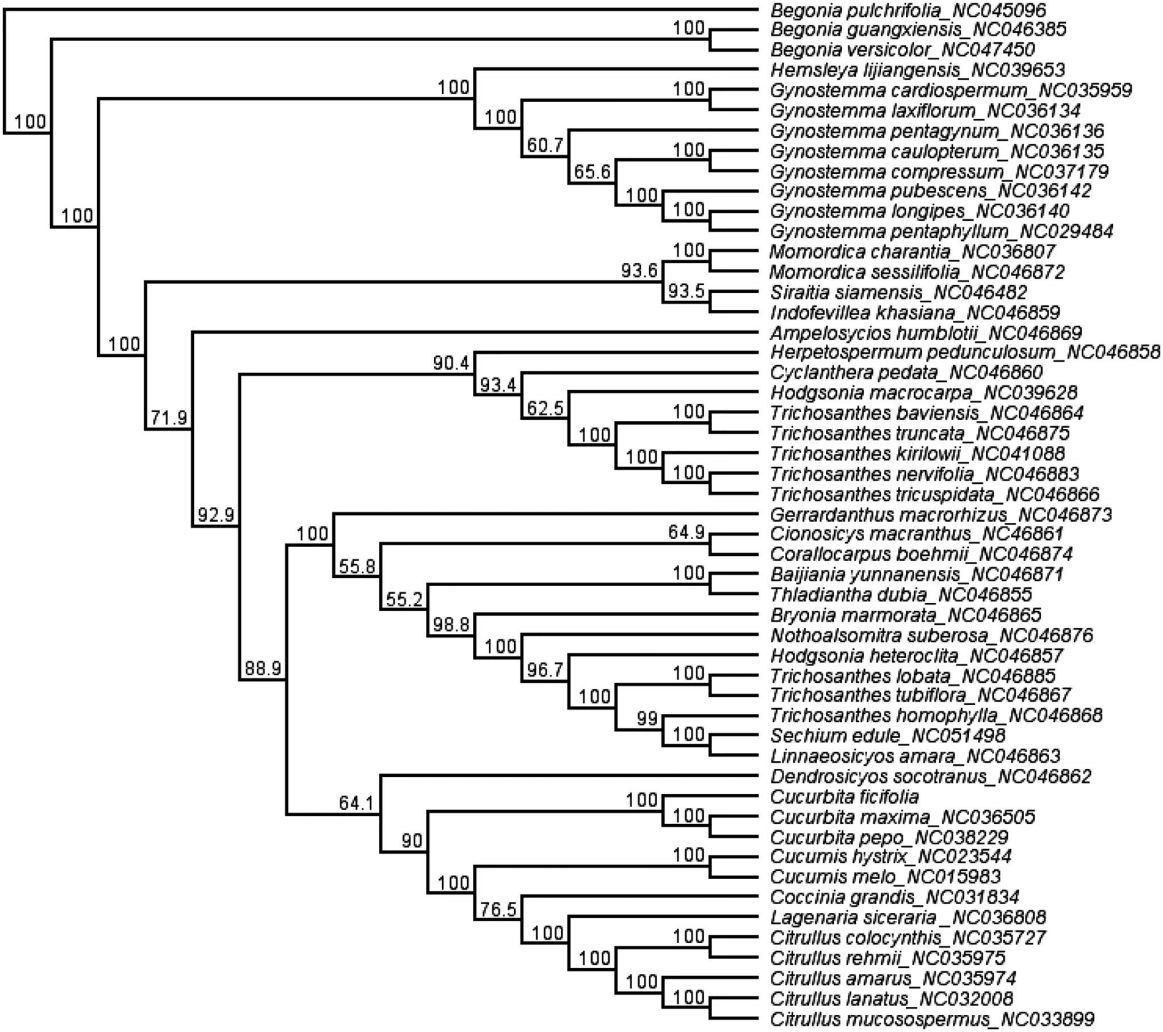
Phylogenetic tree reconstructed by maximum likelihood (ML) analysis based on chloroplast genome sequences, including *Cucurbita ficifolia* sequenced in this study. Numbers below or above branches are assessed by ML bootstrap.

## Data Availability

The genome sequence data that support the findings of this study are openly available in GenBank of NCBI at (https://www.ncbi.nlm.nih.gov/) under the accession no. MW801448. The associated BioProject, SRA, and Bio-Sample numbers are PRJNA716427, SRR14055461, and SAMN18435723, respectively.
